# Engineering FRET biosensor of TDP‐43 to probe formation of TDP‐43 pathology in vitro

**DOI:** 10.1002/alz70855_107363

**Published:** 2025-12-24

**Authors:** Han Zhao, Haowei Xu, Junkai Xie, Shichen Wu, Chongli Yuan

**Affiliations:** ^1^ Purdue University, West Lafayette, IN, USA; ^2^ Purdue University, West lafayette, IN, USA

## Abstract

**Background:**

Alzheimer's disease and related dementias (ADRD) affect 6.2 million people in the U.S., a number that is rapidly increasing due to an aging population, yet no effective cure or treatment exists. TDP‐43 pathology, initially identified as a biomarker for ALS and FTD, is now observed in the majority of ADRD patients and is often associated with accelerated cognitive decline. However, the molecular mechanisms by which TDP‐43 contributes to ADRD progression remain unclear, largely due to the lack of tools to monitor its aggregation dynamics in living cells.

**Methods:**

To address this gap, we engineered a FRET‐based biosensor to detect TDP‐43 aggregation. The biosensor was designed using various TDP‐43 isoforms (full‐length, C‐terminal domain, and ΔNLS) fused to different linker constructs. We evaluated biosensor performance under proteostasis stress conditions known to induce TDP‐43 aggregation and validated its sensitivity using preformed TDP‐43 fibrils. To further explore the progression of TDP‐43 pathology, we transduced the biosensor into human iPSC‐derived cortical neurons. These neurons were then treated with cellular stressors that mimic aging‐related proteostasis dysfunction, cellular stressors that mimic aging‐related proteostasis dysfunction, i.e. preformed fibrils and pharmacological drugs. FRET signal growth was monitored over time, and neurons exhibiting high FRET signals were isolated using FACS for downstream RNA‐seq analysis.

**Results:**

The engineered FRET biosensor successfully detected TDP‐43 aggregation in response to proteostasis stressors. Neurons treated with aging‐mimicking compounds exhibited an increase in FRET signal over time, indicating progressive TDP‐43 aggregation.

**Conclusion:**

This study presents a novel FRET‐based biosensor for tracking TDP‐43 aggregation in living neurons. Our approach enables real‐time monitoring of TDP‐43 pathology and facilitates the identification of molecular pathways disrupted by its accumulation.

